# Apple Pollination: Demand Depends on Variety and Supply Depends on Pollinator Identity

**DOI:** 10.1371/journal.pone.0153889

**Published:** 2016-05-06

**Authors:** M. P. D. Garratt, T. D. Breeze, V. Boreux, M. T. Fountain, M. McKerchar, S. M. Webber, D. J. Coston, N. Jenner, R. Dean, D. B. Westbury, J. C. Biesmeijer, S. G. Potts

**Affiliations:** 1 Centre for Agri-Environmental Research, School of Agriculture, Policy and Development, University of Reading, Reading, United Kingdom; 2 Faculty of Environment and Natural Resources, University of Freiburg, Freiburg, Germany; 3 East Malling Research, East Malling, United Kingdom; 4 Institute of Science & the Environment, University of Worcester, Worcester, United Kingdom; 5 Avalon Produce, Kent, United Kingdom; 6 Naturalis Biodiversity Centre, Leiden, The Netherlands; University of Tsukuba, JAPAN

## Abstract

Insect pollination underpins apple production but the extent to which different pollinator guilds supply this service, particularly across different apple varieties, is unknown. Such information is essential if appropriate orchard management practices are to be targeted and proportional to the potential benefits pollinator species may provide. Here we use a novel combination of pollinator effectiveness assays (floral visit effectiveness), orchard field surveys (flower visitation rate) and pollinator dependence manipulations (pollinator exclusion experiments) to quantify the supply of pollination services provided by four different pollinator guilds to the production of four commercial varieties of apple. We show that not all pollinators are equally effective at pollinating apples, with hoverflies being less effective than solitary bees and bumblebees, and the relative abundance of different pollinator guilds visiting apple flowers of different varieties varies significantly. Based on this, the taxa specific economic benefits to UK apple production have been established. The contribution of insect pollinators to the economic output in all varieties was estimated to be £92.1M across the UK, with contributions varying widely across taxa: solitary bees (£51.4M), honeybees (£21.4M), bumblebees (£18.6M) and hoverflies (£0.7M). This research highlights the differences in the economic benefits of four insect pollinator guilds to four major apple varieties in the UK. This information is essential to underpin appropriate investment in pollination services management and provides a model that can be used in other entomolophilous crops to improve our understanding of crop pollination ecology.

## Introduction

Insect pollination is a key ecosystem service for agriculture, influencing the productivity of ~75% of crop species [1] and contributing ~$361bn to global crop markets in 2009 [2]. The area of insect pollinated crops has grown substantially in recent decades, resulting in greater demands for pollination services [3]. In the UK, evidence suggests that supplies of pollination services, both from managed honeybees [4] and wild pollinators [5,6], do not match these increasing demands. Of the insect pollinated crops grown in the UK, apples (*Malus domestica*) are among the most valuable per hectare and as a self-incompatible crop which requires pollen from other compatible varieties (known as pollinisers) to set fruit, insect pollination services are essential to attaining profitable yields in apples [7]. Garratt *et al*. [8] recently demonstrated that by affecting both the quality and quantity of apples produced, pollination services underpinned ~65% of market output per hectare in two important apple varieties (Cox and Gala).

Managed European honeybees (*Apis mellifera*) can be used as pollinators in large commercial orchards to improve productivity [9,10]. A number of wild insects are also thought to be significant pollinators [11–14]. Notably, mason bees (e.g. *Osmia* spp.), mining bees (e.g. *Andrena* spp.) and bumblebees (*Bombus* spp.) have all been demonstrated to be effective pollinators of apples and, in some cases, more effective than honeybees [12,15–17]. Surveys of pollinator communities visiting UK Cox apple orchards suggest that wild pollinators form the majority of visitors [18], however, there has not been a systematic assessment of the relative pollination service contribution made by different pollinator guilds to apple orchards, or an estimation of the relative economic benefits of different pollinating taxa.

There are examples where crop pollination services do not meet the demand of the crop, resulting in yield and quality deficits [19–21] and sparking interest in the possible economic benefits of increasing pollinator populations. Previous research has shown that outputs in UK Gala orchards could be limited by sub-optimal pollination by ~£6,500/ha [8]. Therefore, improving pollinator management in this crop could provide significant economic returns. How pollinator dependence and possible yield deficits vary between different crop varieties is a fundamental question when considering the economic benefits and management of crop pollinators. Impacts of variety on pollination has only been investigated in a few crops including oilseed [22], blueberry [23] and strawberries [24].

In order to sustainably intensify crop production and meet growing global food demands, it is essential to understand the influence of ecological functions on yield [25]. For insect pollinated crops such as apples, this includes quantifying the impacts of insect pollination services and identifying which species are the most important service providers so they can be appropriately protected and managed. Few studies have considered how crop variety affects dependence on insect pollination or indeed how crop variety affects visitation by different pollinators. Furthermore, although the economic benefits of insect pollinators to crop production have been estimated many times, few studies have estimate the relative economic benefits of different taxa to a single crop. In order to assess the relative importance of different pollinators to different varieties of a crop, this study utilises a combination of pollinator effectiveness measures, visitor observational data and measures of crop dependency to evaluate the supply of pollination service provided by four major pollinator guilds (honeybees, bumblebees, solitary bees and hoverflies) to four UK apple varieties (Cox, Gala, Bramley and Braeburn). In doing so we have: (1) quantified the relative effectiveness of four pollinators to a major UK crop; (2) provided a unique appraisal of the variation in pollination service supply provided by different pollinator guilds across four varieties of a single crop; (3) quantified the demand for insect pollination services of these varieties; and (4) estimated the economic benefits of each pollinator guild to UK production of each variety.

## Materials and Methods

### Pollinator effectiveness

To compare the ability of different pollinators to pollinate apple flowers, both pollinators and apple trees were manipulated using insect flight cages. Four potential pollinators were chosen: the honeybee (*Apis mellifera*), a bumblebee (*Bombus terrestris-audax*), a solitary mason bee (*Osmia bicornis*) and a hoverfly (*Episyrphus balteatus*) and their ability to pollinate *Malus domesticas* var. Scrumptious was studied. This variety was selected because a smaller, potted variety was necessary for use in cage studies. As apples are self-incompatible, a donor variety, Evereste, was also present in all cages. These pollinators represent four distinct flower visiting insect guilds which may provide important pollination services in apple orchards [18]. To manipulate trees and pollinators, insect-proof flight cages were constructed at the University of Reading and University of Leeds experimental farms, using 2.4 x 2.1m frames covered with a polyethylene mesh with a gauge size of 1.33mm. During experiments, each pollinator species was housed in separate flight cages. Each pollinator was provided with appropriate nesting and forage resources within the flight cage when not directly involved in experiments, thus encouraging natural behaviour for the period of experimentation. The honeybees, through the use of a double entrance hive, were given access both to the flight cage and the outside, which could be controlled as needed.

Study trees (variety: Scrumptious and variety: Evereste) were kept in 25L pots. During experiments (spring 2012 and 2013) trees were 2.5 and 3.5 years old respectively and fully productive. When in flower, but not directly involved in experiments, the trees were stored inside isolation flight cages to avoid any interaction with potential pollinators.

Pollination experiments involved placing two flowering polliniser trees (Evereste) into flight cages with each of the four pollinator species. The experiment began when a single apple tree (Scrumptious) was placed in the flight cage. This experimental apple tree was then observed continuously and any insect visits to flowers were recorded by marking a dot on the petal of flowers which received individual visits. This was continued until at least three flowers on that apple tree had received five visits. Each flower which had received a visit was marked with a coloured cable tie; different colours were used to denote flowers which had received a different number of visits (between one and five). The total number of flowers which received each visit number was recorded for each tree. The tree was then stored in an isolation cage until fruit harvest. Pollination experiments were carried out at the end of April and beginning of May in 2012, and mid-May in 2013. The availability of flowering apple trees, polliniser trees and active pollinators enabled 18 trees to be pollinated by bumblebees, 11 by honeybees, three by hoverflies and 13 by solitary bees with a total of 1,831 flowers involved in the study.

In September of each experimental year when apples were ripe, a fruit set measurement was taken. The number of fruit remaining on each tree for each visit number and the original number of flowers which received that number of visits was used to calculate a percentage fruit set for each visit number per tree. During apple development, to prevent damage to trees, non-experimental apples and a small number of experimental fruit were removed from heavily laden branches. Size (max width cm), weight (g) and seed number per apple was measured.

### Pollinator visitation

To compare the flower visitation of different pollinators to different apple varieties, we combined data from a number of UK apple pollinator surveys. Surveys were carried out in Cox, Bramley, Braeburn and Gala orchards in the top fruit growing region of Kent, UK between 2011 and 2014. The owners of the orchards from which data was collected gave permission to conduct the study on these sites. All surveys were carried out in conventionally managed orchards, of varying tree age, surrounded by plantations of other varieties of apple and varying amounts of semi-natural habitat. Orchards of different apple varieties were distributed across data sets and different varieties were often sampled on the same farms, therefore we anticipate no confounding effects of location on pollinators observed visiting flowers of different varieties. Honeybees were not typically utilised for pollination in the orchards although five hives were located close to one of the Gala orchards involved in the surveys. Surveys involved stationary tree observations or mobile transects within the orchards depending on the study (Table A in S1 File). Visitors to apple flowers were recorded to broad taxonomic groups and for transect surveys, where possible, caught and taken back to the laboratory for identification to species.

### Pollinator dependence

To measure the dependence of apple production on insect pollination, three Bramley and two Braeburn orchards were used for experimental trials in 2013. Bramley is the most common variety of culinary apple grown in the UK, accounting for >90% of planted culinary apple area [26]. Braeburn is the third most widely grown dessert apple variety after Cox and Gala, with over 500ha planted as of 2012 [27]. The owners of the orchards from which data was collected gave permission to conduct the study on these sites. Within each of the orchards, three centrally located rows were selected, and on those rows, 10 trees at least 25 m from the orchard edge were involved in the study. Shortly before flowering, two branches on each tree were selected and randomly assigned to one of two treatments: an open treatment and a pollinator exclusion treatment. The pollinator excluded branches were covered with a PVC mesh bag with a mesh size of 1.2 mm^2^ which are wind and rain permeable, but exclude visitation by insects. The number of flowers receiving each treatment was then recorded. When flowering had finished at all sites, bags were removed and the branches were marked with coloured cable ties and string so they could be located for harvest.

Prior to commercial thinning carried out in the orchards (early July), a visit was made to each site. For each branch, the number of set apples was recorded. The apples on each branch, which included any experimental inflorescences, were then thinned according to standard industry practice whereby apples from experimental inflorescences were removed so no more than two remained on any one inflorescence. At the end of the season, all apples from experimental inflorescences were collected one day to a week before commercial harvest (early September for Bramley and late October for Braeburn). Apples were bagged individually by treatment, tree, row and orchard and taken back to the laboratory for quality assessment. Industry standard quality measures for classifying apples for market were taken for all apples collected.

Seed number and maximum width of each apple was recorded. Apples were then scored for shape, either classified as ‘normal’ or ‘deformed’ if there was any shape irregularity. To calculate the economic benefits of pollination to each variety, apples were classed using parameters utilised in the industry (Jenner, 2014 pers. comm.). Apples were classified as class 1 or 2 based on size and shape. Class 1 Braeburn apples are those with no shape deformities and a width greater than 60 mm. Class 1 Bramley apples are between 80–100 mm wide and all other sizes were class 2.

Using the same methodology, the dependence of Cox and Gala apples, and the resultant economic contribution of pollination to profit, had been established in a previous study [8]. These data are analysed in conjunction with data on Bramley and Braeburn for the subsequent economic analysis and pollinator contribution estimates. Data for all four varieties are presented together for the remainder of the manuscript.

### Economic analysis

The economic benefits of pollination services to producers were calculated for each variety following the methods in Garratt et al. [8] by comparing fruit set and quality after commercial thinning, from open pollinated and pollinator excluded treatments. For each treatment, the estimated monetary output of apples produced (£/ha) was calculated with respect to two commercial quality classes using average weekly prices for 2012 from DEFRA [28]. Differences in labour costs, the only cost factor expected to vary by yield, were estimated as the percentage change in the number of apples produced in each treatment multiplied by industry standard costs (Jenner, 2013 pers. Comm.). The impacts of pollination services on output are therefore the differences in the output of apples, less the differences in labour costs from the two treatments (both £/ha). The estimated net change in output was extrapolated to a national scale using the 2012 area of Braeburn and Gala reported in DEFRA [27] and the 2012/2013 area data from DEFRA [26] for Cox and Bramley. In this manuscript we also update the estimated economic benefits of pollination services to Cox and Gala apples reported in Garratt et al. [8] by using 2012 area data alongside 2012 prices. For completeness, results for Gala, Cox, Braeburn and Bramley are reported together for the remainder of the manuscript.

### Pollinator contribution

The contribution of different pollinator guilds to Bramley, Braeburn, Gala and Cox production in the UK was calculated by incorporating pollinator effectiveness, pollinator visitation in the field and the economic benefits of insect pollination to each variety of apple. The effectiveness (E) of each pollinator guild (*i*) was estimated based on a product of the fruit set (F) and seed set (S) resulting from three visits by the taxa to apple flowers in the cage study. Three visits were chosen given that, in the field, apple blossoms can expect a varying number of floral visits and previous research has shown that assuming an apple blossom is receptive for approximately three days and pollinators may be most active for about 6 hours on those days, between two and three visits per flower is a realistic number of visits that one blossom may receive from these pollinators [18]. Given the significant interactive effect of visit number on the pollination effectiveness of our pollinator guilds we also carried out the same economic assessment assuming pollination effectiveness following a single visit. This may better reflect pollinator contributions in years with low overall visitation rates to flowers (Table B in S1 File). The relative pollination service contribution (*R*) of each guild to each variety (v) was calculated as the effectiveness of each guild, multiplied by the observed visitation rate of all members of the guild (T) divided by the effectiveness and visitation rate of all observed pollinators. The standard deviation of the relative pollination service contribution across all sites was taken as a measure of variance.

$$R_{ic}=\frac{\left(E_{iv}\times T_{iv}\right)}{\sum_{i=1}^{i}(E_{v}\times T_{v})}$$

Where *E*_*i*_ = (*F*_*i*_ × *S*_*i*_)

This percentage was then used to calculate the monetary contribution of each pollinator (*GP*) to each apple variety based on the economic benefits of insect pollination to each variety (*PB*).

$$GP_{i}=R_{i}\times PB_{v}$$

As *Bombus terrestris*, *Osmia bicornis* and *Ephyserphis balteatus* may not be representative of the effectiveness of their pollinator guilds as a whole, the economic analysis was re-conducted using only the relative visitation rates of the guild (GT) to each variety without weighting visits by the pollination service effectiveness (Table C in S1 File).

$$GT_{iv}=T_{iv}\times PB_{v}$$

### Statistical analysis

Pollinator effectiveness was analysed using generalised linear mixed effects models to understand effects of pollinator and visit number (1–5) on fruit set and seed set in Scrumptious apples. Pollinator, visit number and their interaction were included in the model as fixed effects; year (2012, 2013), location (Reading, Leeds) and tree were random effects. Fruit set is a proportional response thus a binomial error structure was specified, and seed set is a count so a Poisson error structure was used. Apple width and weight were normally distributed and analysed using linear mixed effects models with the same fixed and random effects as for fruit set and seed number.

Orchard pollinator visitation data were analysed using a generalised linear mixed effects model with pollinator guild (honeybee, bumblebee, hoverfly, solitary bee and other), apple variety (Cox, Gala, Bramley and Braeburn) and a pollinator:variety interaction as main effects in the model. The number of pollinators observed visiting flowers on any given survey day was summed for the analysis so the response variable was a count and thus a Poisson error distribution was defined. Data set, year, survey round and site were included in the model as random effects. An observer level random effect was also included to account for overdispersion. A significant pollinator:variety effect was found so each variety was analysed separately using the same generalised linear model with appropriate random effects as necessary for each data set. Again an observer level random effect was used to account for overdispersion. A Tukey comparison from the ‘multcomp’ R package was used to investigate significant differences between pollinator guilds within varieties.

The dependence of different varieties on insect pollination was analysed using generalised linear mixed effects models to investigate pollination treatment effects on fruit set and seed number. Pollination treatment (open and pollinators excluded) was a fixed effect with tree, nested within row, nested within orchard as random effects. Seed number and fruit set had a Poisson and binomial error structure defined, respectively. A linear mixed effects model with the same fixed and random effects as for the generalised linear mixed effects model was used to analyse apple width. Braeburn width was transformed before analysis. All statistical analysis was carried out in R version 3.2.2.

## Results

### Pollinator effectiveness

**Fig 1:**
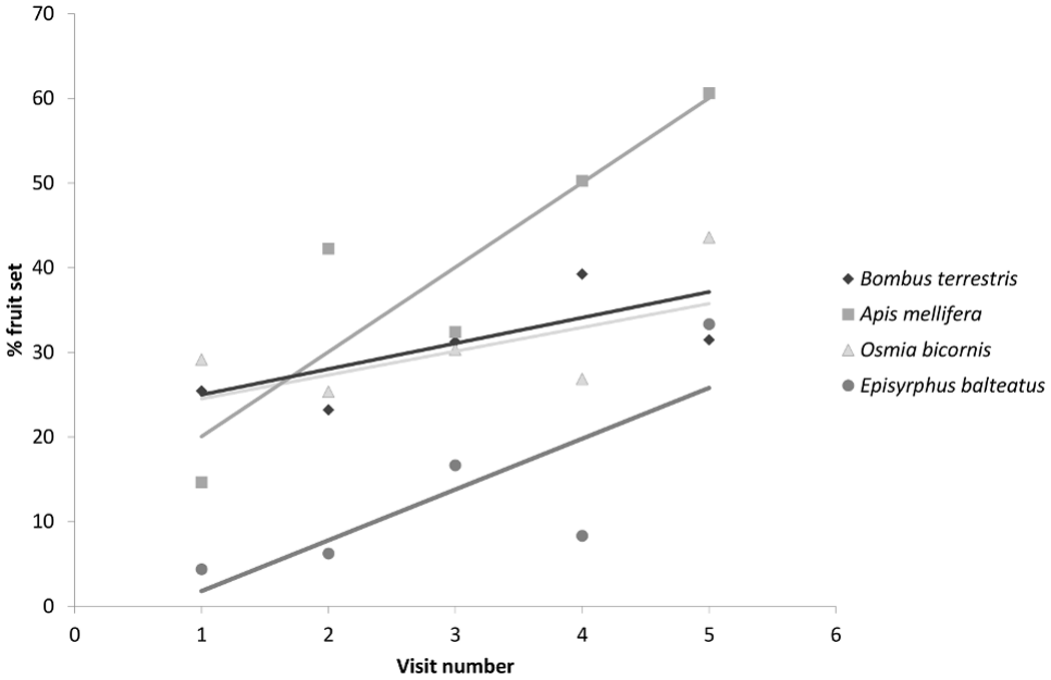
Percentage fruit set of apples (var Scrumptious) following different per flower visit numbers by four pollinator species.

Significant effects of pollinator, visit number and a pollinator:visit number interaction were found on fruit set of experimental apple trees. Fruit set was significantly increased with an increasing number of visits (Z_1,225_ = 2.50, P = 0.01) and *E*. *balteatus* resulted in significantly lower fruit set than *B*. *terrestris* and *O*. *bicornis* (Z_1,225_ > 2.19, P < 0.05). A significant pollinator:visit number interaction (F_3,225_ = 2.65, P = 0.047) indicated that fruit set was more affected by visitation rate of honeybees than for other pollinators (Fig 1). There was a significant effect of pollinator and visit number on seed set per apple. Seed set increased with increasing visit numbers (Z_1,568_ = 2.24, P = 0.025) and *E*. *balteatus* (2.8 ± 2.2) resulted in significantly fewer seeds per apple compared with *B*. *terrestris* (5.1 ± 0.72), *A*. *mellifera* (5.8 ± 0.45) and *O*. *bicornis* (5.6 ± 0.37) (Z_1,568_ > 4.24, P < 0.001). There were no significant pollinator:visit number interactions (F_1,568_ = 0.44, P = 0.72).

There were no significant effects of pollinator, visit number or pollinator:visit number interaction on apple width (*A*. *mellifera* [68.9 ± 1.3], *B*. *terrestris* [63.1 ± 2.9], *O*. *bicornis* [66.8 ± 1.9], *E*. *balteatus* [71.6 ± 3.1]) (pollinator: F_3,35_ = 0.80, P = 0.50; visit number: F_1,460_ = 0.20, P = 0.66; pollinator:visit number: F_3,457_ = 0.23, P = 0.87) or apple weight (*A*. *mellifera* [124.5 ± 5.9], *B*. *terrestris* [105.1 ± 11.2], *O*. *bicornis* [117.7 ± 8.7], *E*. *balteatus* [143.5 ± 12.5]) (pollinator: F_3,35_ = 0.78, P = 0.51; visit number: F_1,456_ = 0.18, P = 0.67; pollinator:visit number: F_3,453_ = 0.51, P = 0.67).

### Pollinator visitation

**Fig 2:**
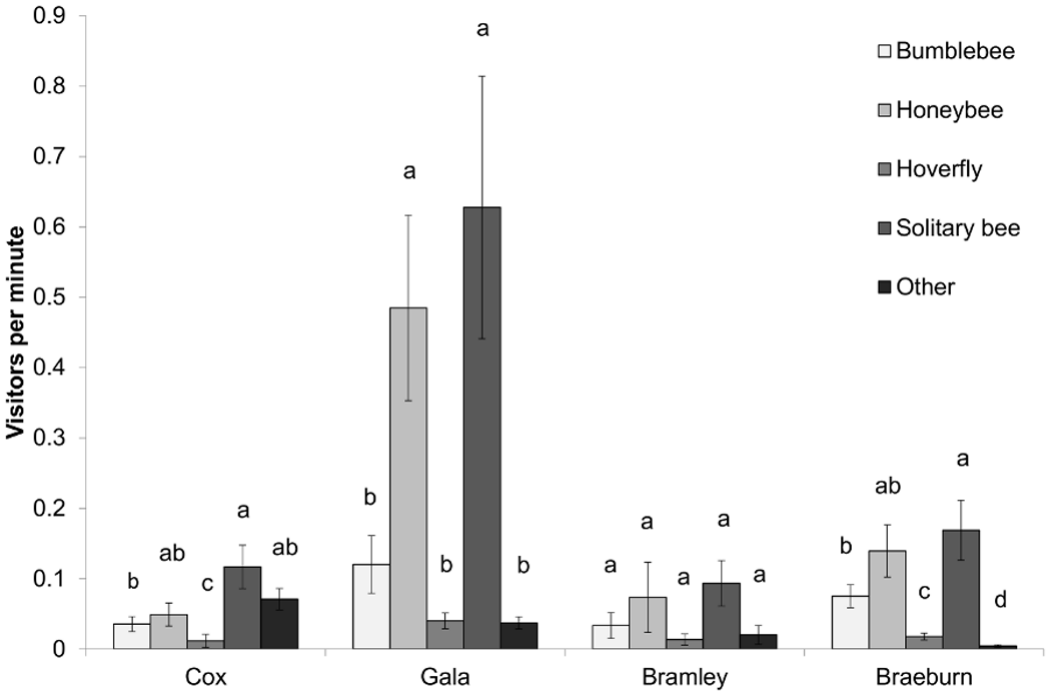
Number of visits observed to different apple variety flowers by different pollinator taxa. Mean ± SE visits per minute per survey shown. Within variety, bars with different letter are significantly different (P < 0.05) following analysis of raw count data using generalised linear mixed effects models.

In the orchards, 1897 insects were observed on apple blossoms: 631 honeybees, 243 bumblebees, 823 solitary bees, 76 hoverflies and 142 other, mostly Diptera individuals. Apple variety affected the pollinator community observed visiting flowers in orchards. When all varieties of apple were included in the analysis there was a significant effect of pollinator (F_4,445_ = 35.25, P < 0.001) and a pollinator:variety interaction (F_12,445_ = 4.26, P < 0.001) on visitation. No significant effect of variety on overall visit number was observed (F_3,445_ = 0.55, P > 0.05). When apple varieties were analysed separately, Cox (F_4,80_ = 9.08, P < 0.001), Braeburn (F_4,240_ = 26.49, P < 0.001) and Gala (F_4,100_ = 10.89, P < 0.001) showed significant effects of pollinator on the number of visits observed, Bramley (F_4,24_ = 2.15, P > 0.05) did not. In Cox orchards, solitary bees were observed visiting flowers significantly more than bumblebees and hoverflies. Hoverflies were also significantly less abundant than all other taxa. In Braeburn, solitary bees were the most abundant followed by honeybees. Bumblebees were also significantly more abundant than hoverflies and ‘other’ visitors. In Gala, solitary bees and honeybees were significantly more abundant than all other taxa (Fig 2).

### Pollinator dependence

**Fig 3:**
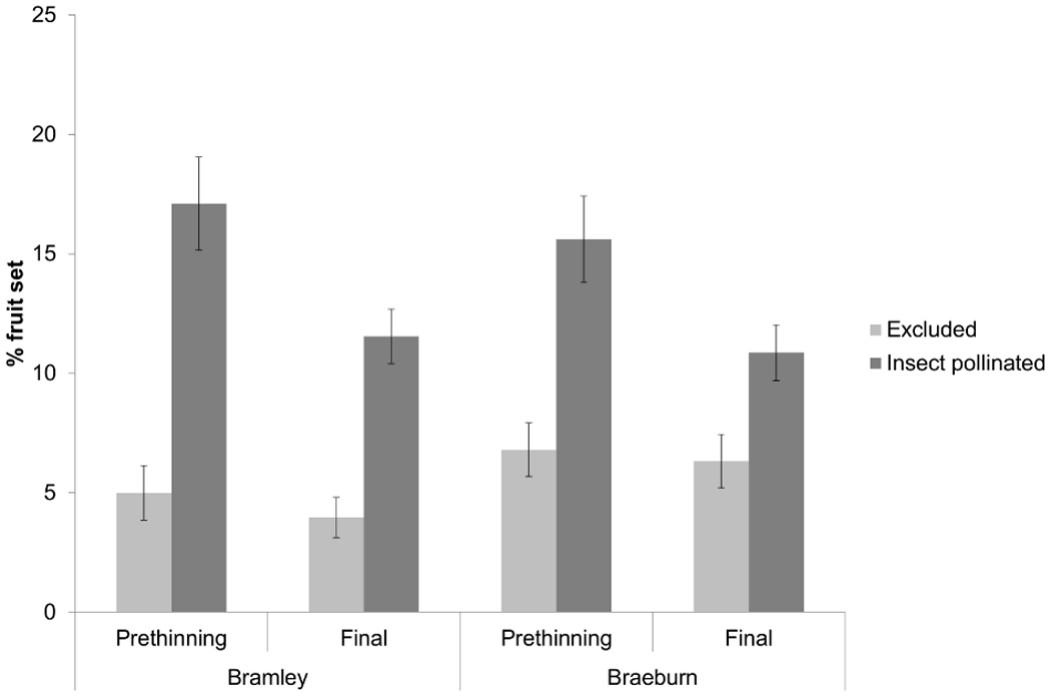
Percentage fruit set pre and post apple thinning for Bramley and Braeburn apples following pollinator exclusion treatments (Mean ± SE).

Pollinator exclusion significantly affected fruit set in both Bramley and Braeburn orchards both before apple thinning (Bramley: Z_1,175_ = 9.33, P < 0.001; Braeburn: Z_1,94_ = 6.14, P < 0.001) and at harvest (Bramley: Z_1,175_ = 7.08, P < 0.001; Braeburn: Z_1,94_ = 3.74, P < 0.001) (Fig 3). With a mean width of 97.0 (SE ± 0.9) cm compared with 93.5 (SE ± 3.4) cm, insect pollination significantly increased Bramley apple size (F_1,22_ = 8.61, P = 0.008). No such significant effect was seen in Braeburn apples, for which mean widths of 68.8 (SE ± 0.3) cm and 67.5 (SE ± 2.7) cm for open and pollinator excluded apples, respectively, were found (F_1,31_ = 3.55, P > 0.05). The number of seeds per apple was significantly affected by pollination treatment for both Bramley (Open [2.2 ± 0.3], Pollinators excluded [0.03 ± 0.03]) (Z_1,193_ = 4.63, P < 0.001) and Braeburn (Open [4.7 ± 1.2], Pollinators excluded [1.3 ± 0.4]) (Z_1,160_ = 9.31, P < 0.001) with seed number in the open treatment greater than in the pollinator exclusion treatment.

### Economic analysis

Analysis of the economic benefits of pollination services indicates that the economic impact of insect pollination on producer profits was £14,500 per hectare for Bramley, £8,500 for Braeburn, £12,300 for Cox and £14,800 for Gala (Table 1). In total, the findings from this study and from the updated findings of Garratt et al. [8] indicate that insect pollination adds £92.1M to UK apple production for these four varieties.

**Table 1:** Summary of the economic benefits of pollination services to UK Apple varieties in 2012.

	Cox	Gala	Bramley	Braeburn
Area (ha)	1,697	1,312	3,326	509
Price/Kg class 1(£)	0.86	0.77	£0.83	£0.85
Price/Kg class 2 (£)	0.50	0.52	£0.53	£0.55
Total benefits/ha (£000)	£20.1	£22.9	£21.2	£18.2
Total IPB/ha (£000)	£12.3	£14.8	£14.5	£8.5
National Total IPB (£000)	£20,214.7	£19,374.3	£48,120.6	£4,339.7

Area = the total area reported in 2012 in the Orchard Fruit Survey (Braeburn/Gala:) and in the crop year 2012/2013 (Cox/Bramley:). Total benefits/ha = the total economic benefits of market output per hectare estimated from the open pollination treatment. Total IPB/ha = the total economic benefits of insect pollination services per hectare; the difference between the value per hectare in the open and closed treatments. National Total IPB = the total economic benefits of insect pollination services to the crop across the UK.

### Pollinator contribution

Based on effectiveness and visitation in the field, solitary bees were found to contribute to more than 50% of pollination service in three of the four varieties studied, Cox, Gala and Bramley. Bumblebees were important pollinators of Braeburn (38% of services) but otherwise accounted for <21% of services in other varieties. Honeybees consistently contributed between 23–28% of pollination services although there was often substantial variation between orchards. Due to their low visitation rates and poor pollination effectiveness, hoverflies contributed less than 3% of pollination to all varieties. Solitary bees had the most consistent presence between orchards and were never totally absent from any orchard studied. By contrast, honeybee and bumblebee presence could vary greatly depending on the variety and between orchards (Table 2).

**Table 2:** Estimated pollination services and economic benefits to each variety provided by the four pollinator guilds studied based on visitation rates and effectiveness (after 3 visits). Measures of standard deviation are included in brackets.

Pollinator	Variety								
	Cox		Gala		Bramley		Braeburn		
	Proportion of Service (%)	Benefit (£M)	Proportion of Service (%)	Benefit (£M)	Proportion of Service (%)	Benefit (£M)	Proportion of Service (%)	Benefit (£M)	Total Benefit (£M)
Bumblebees	21% (±13%)	£4.2 (±2.7)M	13% (±19%)	£5.3 (±5.4)M	15% (±17%)	£7.4 (±8.3) M	38% (±33%)	£1.7 (±1.4)M	£18.6M (±£17.8M)
Honeybee	25% (±14%)	£5.1 (±2.8)M	28% (±28%)	£2.6 (±3.6)M	26% (±30%)	£12.7(±14.7)M	23% (±22%)	£1.0 (±0.9)M	£21.4M (± £22M)
Hoverflies	0.3% (±1%)	£0.1 (±0.1)M	2% (±5%)	£0.4 (±1.0)M	0.4% (±1%)	£0.2 (±0.3) M	1% (±1%)	£0.04(±0.06)M	£0.7M (±1.5M)
Solitary bees	54% (±21%)	£10.9 (±4.1)M	57% (±29%)	£11.0 (±5.5)M	58% (±39%)	£27.8 (±18.8)M	39% (±24%)	£1.7 (±1.0)M	£51.4M (±29.4M)

Proportion of service (%) = the average percentage contribution to total pollination services made by the taxa to the variety. Benefits (£M) = the monetary benefits, in million £ of additional production, of the pollination services provided by the taxa to that specific variety.

Extrapolating the results up to a UK scale, solitary bees are estimated to be the most economically valuable guild to the apple varieties studied increasing productivity by £51.4M (±29.4M) while hoverflies contributed the lowest benefits (£0.7M ±1.4M). Honeybees were generally more valuable than bumblebees due to their greater contribution to Bramley and Gala, two widespread varieties. However, the honeybee contribution was also highly variable in these varieties (s.d. ~±29%), resulting in a significant variability in estimated benefits (Table 2).

Estimating the benefits provided by different pollinator guilds based on single visit effectiveness (Table B in S1 File) or their visitation rates alone (Table C in S1 File) has little effect on the ranked contributions, with solitary bees remaining the most important guild in all four varieties nationally. However, the monetary benefits attributed to hoverflies rise substantially (£0.7M-£4.2M nationally).

## Discussion

Solitary bees, honeybees, bumblebees and hoverflies can all pollinate apples, although hoverflies were shown to be the least effective of the taxa studied. Pollinator visitation in orchards is significantly affected by apple variety and some pollinator guilds are more active on some varieties than others. This could be a result of varying nectar and pollen availability between apple varieties [29]. Using a combination of field observations and cage experiments, this study highlights the variations in relative service contribution made by four major pollinator guilds across four different varieties; this contribution is a combination of their pollination effectiveness for apples and flower visitation rates in commercial orchards, as well as the dependence of these varieties on insects for pollination. The findings further demonstrate the economic benefits of insect pollination services to UK apple orchards, estimating economic benefits to producers of ~£92M across the four varieties studied.

The differences found between pollinator guilds and their contribution to the production of different varieties, despite spatial and temporal overlap in the surveys, indicate some varieties are better serviced by some pollinators than others. Management to maintain or enhance pollinator populations could therefore be targeted for particular varieties. Given their proven capacity to pollinate apples, as demonstrated in this study and others [10], management involving introduction of honeybees may provide a potential solution to maintain or improve apple pollination. Historically, honeybees have been widely utilised for their pollination services in UK orchards [30] but at present it remains unknown how widespread this practice is and careful management is essential to prevent honeybees from engaging in sub-optimal foraging [10,31]. The highly variable contribution made by honeybees to pollination service in some varieties suggests their utilisation could be extended. Findings from this research could guide appropriate remuneration for apiculturists providing hives for pollination services in UK apples.

This research shows that currently the majority of the pollination service to apples in the UK is provided by wild pollinators (£70.7M p.a.) rather than managed honeybees (£21.4M p.a.), with solitary bees in particular making a large contribution (£51.4M p.a.), both through their capacity to pollinate apple flowers effectively and flower visitation frequency. Management to increase wild pollinators often takes time to establish and produce effects. The perennial nature of apples makes local and wider landscape pollinator management practices more appropriate than in annual rotation crops, particularly given the time it takes for mitigation measures such as establishment of flower strips or altered management practices to benefit and build up wild pollinator populations. Such management will result in returns on the initial investment over the lifespan of the tree crop which can often be up to 20 years. Such returns on investment in pollinator management strategies have been demonstrated in blueberry crops [32]. Wild bees require additional nectar and pollen and so planting wildflower strips in orchards can increase the abundance and reproductive success of flower visiting solitary bees [33]. Furthermore, establishment and preservation of semi-natural habitat consistently increases the diversity and abundance of wild pollinators [34] and more specifically, increased woodland habitat can benefit solitary bees in apple orchards [12,35]. Similarly, providing additional artificial nesting resources can boost solitary bee populations and improve pollination service [36–38]. Such management practices could be implemented across apple varieties, all of which are heavily reliant on solitary bees. The £51.4M contribution solitary bees make to these varieties in the UK alone, highlights the potentially serious financial implications of any declines in these species and emphasises the need for effective management strategies. The relatively large contribution bumblebees make to Braeburn pollination (38%) could warrant focused management on these species in and around Braeburn orchards. Planting pollen and nectar rich species can increase local bumblebee abundance and species richness [39] while field boundaries can provide suitable nesting sites for many bumblebees [40]. Undertaking both these measures could therefore be an effective means of boosting pollination service in the long term. Increasing wild pollinator populations provides additional benefits associated with a diverse pollinator assemblage including service resilience, insurance for inter-annual variation and complimentarily [41–43]. However further work will be required to assess the cost effectiveness and co-benefits of any such management plan (e.g.[32]).

As with a number of previous studies, estimates of the economic benefits of pollination services are limited by the assumption of constant prices and the potential complexities of extrapolating impacts from smaller scales up to a national level [44]. In particular, the benefits reported here may vary depending on the presence of other inputs or ecosystem services [45]. The benefits estimated only reflect current benefits to producer profits rather than wider societal impacts (i.e. economic value); in the event of a collapse of pollination services, the benefits lost would be substantially different as prices respond and producers substitute their inputs to compensate [46]. As such these findings may over- or under-estimate the actual impacts of pollination. However, as the majority of UK apple consumption is imported [26] and there is little to indicate that imports could not be increased, the impacts on consumers are likely to be negligible. As such, despite some limitations, the economic benefits estimated in this study are likely to be the most accurate currently available.

Using combined findings from cage experiments, pollinator surveys and field manipulations, this study quantifies the contribution of different pollinator guilds to UK apple production which represents a significant step forward but, to do this, several assumptions have been made. In the first instance, a single pollinator species was used as a surrogate to measure and represent the pollination effectiveness of a pollinator guild but clearly the pollinator community visiting apple orchards is diverse (Table D in S1 File). In the case of *Apis mallifera* this is entirely appropriate as no other honeybee species are found in the UK. However other guilds are more diverse. This analysis makes the assumption that pollinator effectiveness is more similar within pollinator guilds than between pollinator guilds and, considering factors which will influence the effectiveness of pollinators when visiting flowers, including morphology, body size and pollen collecting habit, there is some justification for this assumption. For instance, *Osmia sp*. and *Andrena sp*. store pollen using scopae unlike corbiculate guilds like the bumblebees and honeybees. Also the solitary bees observed in our study orchards are all smaller than UK bumblebees. Furthermore, hoverflies will forage only for nectar and not pollen. The use of relative pollination effectiveness in the analysis rather than absolute pollination effectiveness minimises the risk that conclusions drawn for one species do not reflect the pollinator guild as a whole. Despite the limitation of using a surrogate species to represent a pollinator guild, including a measure of effectiveness rather than visitation alone improves our estimate of pollinator contributions.

Re-estimating the economic benefits provided by each guild without weighting for pollinator effectiveness indicates that the findings change only moderately with the exception of an increase in the benefits attributed to hoverflies (Table C in S1 File) due to the low weighting afforded to their pollination effectiveness based on cage studies. The outcomes of the study would be more highly resolved if pollination effectiveness could be measured for different species within each pollinator guild and linked to visitation rates of those species in the field, but for practical reason, it is not possible to conduct a study of this scale. Nonetheless, this shortcoming highlights the need to determine appropriate proxies for pollination service analysis in future, based on shared traits within a guild.

In the present study, the pollination effectiveness of the four guilds on the variety Scrumptious is taken to represent their pollination effectiveness to apples as a whole. Again, the use of a relative measure of pollinator effectiveness allows for differences between the fruit set of different varieties following insect visitation and, while flower morphology invariably affects the behaviour and effectiveness of flower visiting insects (e.g. [15]), there is little variation in the floral morphology of the apple varieties studied (personal observation). Furthermore, fruit set will be strongly affected by the amount of viable polliniser pollen pollinators are carrying during floral visits. This is itself a product of each guilds visitation rate to polliniser trees and their between tree and between row movement in orchards. It is also affected by the number and distribution of polliniser trees in the orchards, as well as their compatibility with the variety in question [47]. These factors vary hugely between orchards in the UK and therefore findings from the cage experiments in the present study represent accurate relative pollination efficiencies for each of the pollinator guilds, independent of variations in polliniser availability.

This is the first time measures of pollinator effectiveness and field abundance have been combined and compared between pollinator guilds to quantify their contribution to crop production and economic output. It is also the first time that pollinator guild contributions have been compared between different varieties of a crop. As our knowledge of the pollination efficiency of different pollinators to different crops grows and consolidates globally [48], the concepts used in this study can be applied to better quantify economic impacts of different components of the pollinator community on crop production. This can ultimately result in more holistic models of pollination service provision and facilitate better modelling of the risks of pollinator declines [44]. Specifically, this study highlights the significant contribution made by insect pollinators to UK apple production. The variable pollination effectiveness of different pollinator guilds for apples has been demonstrated and when this is combined with flower visitation in the field, the contribution of different pollinator guilds to the production of different apple varieties is pronounced. These findings have implications for the management of insect pollination services in apple orchards and highlight the potential consequences of any decline in specific taxa and advocates management targeted to specific varieties. The £92.1M insect pollinators contribute to apple production in the UK suggests that further investment in the research and implementation of insect pollinator management strategies as part of an integrated orchard management system is justified.

## References

[1] Klein A-M, Vaissière BE, Cane JH, Steffan-Dewenter I, Cunningham SA, Kremen C, et al (2007) Importance of pollinators in changing landscapes for world crops. Proceedings of the Royal Society B: Biological Sciences 274: 303–313. 17164193 10.1098/rspb.2006.3721 PMC1702377

[2] Lautenbach S, Seppelt R, Liebscher J, Dormann CF (2012) Spatial and Temporal Trends of Global Pollination Benefit. PloS one 7: e35954 10.1371/journal.pone.0035954 22563427 PMC3338563

[3] Aizen MA, Garibaldi LA, Cunningham SA, Klein AM (2008) Long-Term Global Trends in Crop Yield and Production Reveal No Current Pollination Shortage but Increasing Pollinator Dependency. Current Biology 18: 1572–1575. 10.1016/j.cub.2008.08.066 18926704

[4] Breeze TD, Vaissière BE, Bommarco R, Petanidou T, Seraphides N, Kozák L, et al (2014) Agricultural Policies Exacerbate Honeybee Pollination Service Supply-Demand Mismatches Across Europe. PloS one 9: e82996 10.1371/journal.pone.0082996 24421873 PMC3885438

[5] Schulp CJE, Lautenbach S, Verburg PH (2014) Quantifying and mapping ecosystem services: Demand and supply of pollination in the European Union. Ecological Indicators 36: 131–141.

[6] Polce C, Termansen M, Aguirre-Gutierrez J, Boatman ND, Budge GE, Crowe A, et al (2013) Species Distribution Models for Crop Pollination: A Modelling Framework Applied to Great Britain. Plos One 8. 10.1371/journal.pone.0076308 PMC3796555 24155899

[7] Ramírez F, Davenport TL (2013) Apple pollination: A review. Scientia Horticulturae 162: 188–203.

[8] Garratt MPD, Breeze TD, Jenner N, Polce C, Biesmeijer JC, Potts SG (2014) Avoiding a bad apple: Insect pollination enhances fruit quality and economic value. Agriculture, Ecosystems & Environment 184: 34–40. 10.1016/j.agee.2013.10.032 PMC3990452 24748698

[9] Delaplane KS, Mayer NF (2000) Crop Pollination by Bees. Wallingford: CABI Publishing.

[10] Stern RA, Eisikowitch D, Dag A (2001) Sequential introduction of honeybee colonies and doubling their density increases cross-pollination, fruit-set and yield in 'Red Delicious' apple. Journal of Horticultural Science & Biotechnology 76: 17–23.

[11] Russo L, Park M, Gibbs J, Danforth B (2015) The challenge of accurately documenting bee species richness in agroecosystems: bee diversity in eastern apple orchards. Ecology and Evolution 5: 3531–3540. 10.1002/ece3.1582 26380684 PMC4567859

[12] Martins KT, Gonzalez A, Lechowicz MJ (2015) Pollination services are mediated by bee functional diversity and landscape context. Agriculture, Ecosystems & Environment 200: 12–20.

[13] Kleijn D, Winfree R, Bartomeus I, Carvalheiro LG, Henry M, Isaacs R, et al (2015) Delivery of crop pollination services is an insufficient argument for wild pollinator conservation. Nat Commun 6. 10.1038/ncomms8414 PMC4490361 26079893

[14] Blitzer EJ, Gibbs J, Park MG, Danforth BN (2016) Pollination services for apple are dependent on diverse wild bee communities. Agriculture, Ecosystems & Environment 221: 1–7.

[15] Matsumoto S, Abe A, Maejima T (2009) Foraging behavior of Osmia cornifrons in an apple orchard. Scientia Horticulturae 121: 73–79.

[16] Thomson JD, Goodell K (2001) Pollen removal and deposition by honeybee and bumblebee visitors to apple and almond flowers. Journal of Applied Ecology 38: 1032–1044.

[17] Vicens N, Bosch J (2000) Pollinating Efficacy of Osmia cornuta and Apis mellifera (Hymenoptera: Megachilidae, Apidae) on ‘Red Delicious’ Apple. Environmental Entomology 29: 235–240.

[18] Garratt MPD, Truslove L, Coston D, Evans R, Moss E, Dodson C, et al (2014) Pollination deficits in UK apple orchards. Journal of Pollination Ecology 12: 9–14.

[19] Brookfield PL, Ferguson IB, Watkins CB, Bowen JH (1996) Seed number and calcium concentrations of 'Braeburn' apple fruit. Journal of Horticultural Science 71: 265–271.

[20] Pritchard KD, Edwards W (2006) Supplementary pollination in the production of custard apple (Annona sp.)—the effect of pollen source. Journal of Horticultural Science & Biotechnology 81: 78–83.

[21] Holzschuh A, Dudenhöffer J-H, Tscharntke T (2012) Landscapes with wild bee habitats enhance pollination, fruit set and yield of sweet cherry. Biological Conservation 153: 101–107.

[22] Hudewenz A, Pufal G, Bogeholz A-L, Klein A-M (2013) Cross-pollination benefits differ among oilseed rape varieties. The Journal of Agricultural Science FirstView: 1–9.

[23] Benjamin FE, Winfree R (2014) Lack of Pollinators Limits Fruit Production in Commercial Blueberry (Vaccinium corymbosum). Environmental Entomology 43: 1574–1583. 10.1603/EN13314 25313694

[24] Klatt BK, Holzschuh A, Westphal C, Clough Y, Smit I, Pawelzik E, et al (2014) Bee pollination improves crop quality, shelf life and commercial value. Proceedings of the Royal Society of London B: Biological Sciences 281. 10.1098/rspb.2013.2440 PMC3866401 24307669

[25] Bommarco R, Kleijn D, Potts SG (2013) Ecological intensification: harnessing ecosystem services for food security. Trends in Ecology & Evolution 28: 230–238. 23153724 10.1016/j.tree.2012.10.012

[26] DEFRA (2014) Basic Horticultural Statistics.

[27] DEFRA (2013) Orchard Fruit Survey.

[28] DEFRA (2014) National Average Wholesale Prices of Selected Home-grown Horticultural Produce, England.

[29] Schneider D, Stern RA, Eisikowitch D, Goldway M (2002) The relationship between floral structure and honeybee pollination eficiency in ‘Jonathan’ and ‘Topred’ apple cultivars. The Journal of Horticultural Science and Biotechnology 77: 48–51.

[30] Carreck NL, Williams IH, Little DJ (1997) The movement of honey bee colonies for crop pollination and honey production by beekeepers in Great Britain. Bee World 78: 67–77.

[31] Mayer DF, Lunden JD (1991) Honey bee foraging on dandelion and apple in apple orchards. Journal of the Entomological Society of British Columbia 88: 15–17.

[32] Blaauw BR, Isaacs R (2014) Flower plantings increase wild bee abundance and the pollination services provided to a pollination-dependent crop. Journal of Applied Ecology 51: 890–898.

[33] Sheffield CS, Westby SM, Smith RF, Kevan PG (2008) Potential of bigleaf lupine for building and sustaining Osmia lignaria populations for pollination of apple. The Canadian Entomologist 140: 589–599.

[34] Garibaldi LA, Steffan-Dewenter I, Kremen C, Morales JM, Bommarco R, Cunningham SA, et al (2011) Stability of pollination services decreases with isolation from natural areas despite honey bee visits. Ecology Letters 14: 1062–1072. 10.1111/j.1461-0248.2011.01669.x 21806746

[35] Watson JC, Wolf AT, Ascher JS (2011) Forested Landscapes Promote Richness and Abundance of Native Bees (Hymenoptera: Apoidea: Anthophila) in Wisconsin Apple Orchards. Environmental Entomology 40: 621–632. 10.1603/EN10231 22251640

[36] Bosch J, Kemp WP (2002) Developing and establishing bee species as crop pollinators: the example of Osmia spp. (Hymenoptera: Megachilidae) and fruit trees. Bulletin of Entomological Research 92: 3–16. 12020357 10.1079/BER2001139

[37] Sheffield CS (2014) Pollination, seed set and fruit quality in apple: studies with Osmia lignaria (Hymenoptera: Megachilidae) in the Annapolis Valley, Nova Scotia, Canada. Journal of Pollination Ecology 12.

[38] Gruber B, Eckel K, Everaars J, Dormann C (2011) On managing the red mason bee (Osmia bicornis) in apple orchards. Apidologie 42: 564–576.

[39] Carvell C, Osborne JL, Bourke AFG, Freeman SN, Pywell RF, Heard MS (2011) Bumble bee species' responses to a targeted conservation measure depend on landscape context and habitat quality. Ecological Applications 21: 1760–1771. 21830716 10.1890/10-0677.1

[40] Kells AR, Goulson D (2003) Preferred nesting sites of bumblebee queens (Hymenoptera: Apidae) in agroecosystems in the UK. Biological Conservation 109: 165–174.

[41] Winfree R, Kremen C (2009) Are ecosystem services stabilized by differences among species? A test using crop pollination. Proceedings of the Royal Society B: Biological Sciences 276: 229–237. 10.1098/rspb.2008.0709 18796401 PMC2674338

[42] Brittain C, Kremen C, Klein AM (2013) Biodiversity buffers pollination from changes in environmental conditions. Global Change Biology 19: 540–547. 10.1111/gcb.12043 23504791

[43] Brittain C, Williams N, Kremen C, Klein A-M (2013) Synergistic effects of non-Apis bees and honey bees for pollination services. Proceedings of the Royal Society Biological Sciences Series B 280: 1–7. 10.1098/rspb.2012.2767 PMC3574329 23303545

[44] Melathopoulos AP, Cutler GC, Tyedmers P (2015) Where is the value in valuing pollination ecosystem services to agriculture? Ecological Economics 109: 59–70.

[45] Melathopoulos AP, Tyedmers P, Cutler GC (2014) Contextualising pollination benefits: effect of insecticide and fungicide use on fruit set and weight from bee pollination in lowbush blueberry. Annals of Applied Biology 165: 387–394.

[46] Bauer DM, Wing S (2014) The Macroeconomic Cost of Catastrophic Pollinators Decline.

[47] Kron P, Husband BC, Kevan PG, Belaoussoff S (2001) Factors affecting pollen dispersal in high-density apple orchards. Hortscience 36: 1039–1046.

[48] Garibaldi LA, Bartomeus I, Bommarco R, Klein AM, Cunningham SA, Aizen MA, et al (2015) EDITOR'S CHOICE: REVIEW: Trait matching of flower visitors and crops predicts fruit set better than trait diversity. Journal of Applied Ecology 52: 1436–1444.

